# Burnout syndrome among Libyan physicians in different specialties: an observational cross-sectional study

**DOI:** 10.3389/fmed.2026.1856607

**Published:** 2026-07-13

**Authors:** Mawadda Faruk Benhamza, Heebah Alhadi Abdulhamid, Boshra Hagi Abusahmin, Mohamed Hadi Mohamed Abdelhamid

**Affiliations:** 1 Research, Documentation, and Scientific Committee Office, National Center for Disease Control (NCDC), Tripoli, Libya; 2 The Department of Community Medicine at the Faculty of Medicine, University of Tripoli, Tripoli, Libya; 3 Libyan Authority for Scientific Research, Tripoli, Libya; 4 Cell Biology and Tissue Culture Department, Libyan Biotechnology Research Center (BTRC), Tripoli, Libya

**Keywords:** burnout, Libya, Maslach burnout inventory, physician, prevalence

## Abstract

Burnout is an occupation-related syndrome characterized by emotional exhaustion (EE), depersonalization (DP), and a diminished sense of personal accomplishment (PA) at work. The healthcare profession is an exhausting job that involves individual health and mental well-being. The aim is to determine the prevalence and severity of burnout syndrome among Libyan physicians. An observational cross-sectional study was conducted from March to September 2023, recruiting 731 physicians from both public and private sectors, encompassing various specialties and subspecialties. Upon agreeing to participate, physicians were assessed in two sections. Section One included socio-demographic characteristics. In section two, burnout was assessed using the Maslach Burnout Inventory-Human Services Survey (MBI-HSS). In our study, 731 physicians from various health facilities participated. The average age was 36.76 years (±7.23). The Sex distribution was 38.9% male and 61.1% female. More than half of the physicians were married (60.7%). The average income was 1,672 LD (approximately US$344.74) with a standard deviation of 2177.19 LD. The mean number of vacation days taken annually was 16.85 days (±26.03). Among the three dimensions, 47.1% of the physicians reported a high score of burnout for EE, 86.3% for depersonalization, and 18.6% for personal achievement. Age, marital status, specialty, and place of work were significant risk factors for burnout in three dimensions; EE, DP, and PA (*p*-value <0.05); the highest burnout rates were 16.8% for the medicine, 10.0% for the ICU and anesthesia, 7.6% for surgery, 5.3% for obstetrics and gynecology, and 4.2% for pediatrics. In conclusion, physician burnout is declared a public health threat if no immediate action is taken to mitigate a work-related burden on physicians.

## Introduction

Burnout syndrome is “a state of mental and physical exhaustion caused by one’s professional life” (1). It involves three main components: emotional exhaustion, depersonalization, and personal accomplishment. In fact, in recent years, burnout syndrome has become one of the most widely discussed mental health problems in modern societies. The concept of burnout, first described by Herbert Freudenberger in 1974, was based on his observations of emotional distress and chronic fatigue among volunteer workers at free clinics for drug addiction in New York City (2). Moreover, to assess burnout prevalence, this study uses the Maslach Burnout Inventory - Human Services Survey (MBI-HSS). Developed by Maslach and Jackson in 1981, the MBI-HSS is a 22-item psychometric instrument that measures burnout across three subscales: emotional exhaustion, depersonalization, and reduced personal accomplishment. Although the original framework focused on professionals in public and human services, the tool has since been adapted into multiple context-specific versions. These versions include: MBI-HSS (MP), designed specifically for healthcare professionals who deal with patients; MBI-ES for educators; and MBI-General Survey (MBI-GS), which adapts the framework for professions that do not involve direct care for clients or patients (3).

In the same vein, the 11th revision of the International Classification of Diseases (ICD) recognized burnout as an occupational phenomenon. However, it is categorized as a factor affecting health status or contact with health services, rather than as a medical disease requiring treatment (4).

Burnout is becoming widespread across all professions; even so, healthcare providers, mainly physicians, are still at the top of the list, regardless of their profession or subspecialty. According to many studies, between 31 and 49.6% of medical students in the United States experienced burnout as of 2010 (5). Also, a 2009 US study found that residents in internal medicine and surgery were impacted by 50 and 76%, respectively (6). Compared to these statistics, in 2021, Medscape reported that 42% of US physicians experienced burnout, and in 2022, the number rose to 47% (7).

In contrast, a 2006 study in Canada by Legassie et al. reported that only 12.5% of medical residents scored positively on all three dimensions of the Maslach Burnout Inventory (8). However, it is important to note here a need to clarify the basic framework to explicitly emphasize that the Maslach Burnout Inventory (MBI-HSS) is not used as a diagnostic tool for individual mental illnesses, but rather measures the severity of a multifaceted occupational syndrome largely resulting from systemic environmental failures.

Furthermore, according to some studies, political instability and active warfare are primary drivers accelerating high levels of emotional exhaustion and overall burnout among physicians in conflict-affected developing nations such as Yemen, Sudan, and Syria (9–11). One of these studies, conducted in Yemen in 2010, surveyed 563 physicians. Overall, 63.2% of the participants had high emotional exhaustion, 19.4% had high depersonalization, and 33% had low personal accomplishment (9). In a 2022 study conducted in Sudan, 208 physicians reported burnout across three dimensions: emotional exhaustion (EE), with 70.7% showing high levels; depersonalization (DP), with 44.2% showing high levels; and professional accomplishment (PA), with 73.1% showing low levels (10).

In addition, a study was performed in Syria (2019) to determine the prevalence of burnout in different specialties by assessing three dimensions of the MBI-HSS scale; the results revealed a high level of EE (77.9%), a high level of DP (54.6%), and a low level of PA (64.5%) (11).

Moreover, in studies with limited sample sizes conducted during the COVID-19 pandemic and the civil war, the prevalence of burnout syndrome among Libyan healthcare workers was high: 67.1% reported experiencing a high level of EE, 47.4% reported a high level of DP, and 22.7% scored low on the PA subscale (12). Similarly, a 2022 study at the Department of Internal Medicine in Benghazi found that physicians experienced high levels of EE at 14.7% and high DP at 92%, while 87.3% of respondents reported low scores for personal accomplishment (13).

The main concern of burnout syndrome among physicians centered on consequences that affect the quality of patients’ lives in terms of decreased performance, decision-making, and management, in addition to the quality of their own lives. It can also increase individuals’ risk of suffering from depression, anxiety, sleep disturbances, fatigue, alcohol and drug misuse, marital problems, premature retirement, and suicide (12). Physician burnout might be worse in developing countries due to a poor working environment and a lack of opportunities for career growth in terms of training programs and payment rates (14).

Maslach used these definitions to develop the Maslach Burnout Inventory (MBI-HSS), which is widely regarded as the most reliable and validated instrument for measuring burnout among doctors (15).

The existing data on burnout syndrome among Libyan physicians is limited and fails to provide a comprehensive understanding of the issue. Therefore, we undertook this study to investigate the underlying factors contributing to burnout within this population. Specifically, this study aims to assess the prevalence, severity, and key determinants of burnout syndrome among Libyan physicians.

## Materials and methods

### Study design and data collection tool

An observational, cross-sectional study design was utilized to evaluate associations between workplace factors and burnout; given the single-time-point nature of data collection, this design focuses on identifying statistical correlations rather than establishing definitive causal pathways. Data collection spanned 5 months from March to September 2023 using a non-probability convenience sampling approach, yielding a final sample of 731 valid responses.

To overcome regional logistical barriers and capture a highly diverse cross-section of the physician workforce, a dual-channel hybrid distribution strategy was deployed. First, the structured electronic questionnaire was hosted on the KoboToolbox platform.^1^ This link was systematically disseminated nationally by local medical associations via their official email distribution lists, verified Viber/WhatsApp professional communication channels, and medical specialty networks. To prevent duplicate submissions and ensure data integrity, the KoboToolbox platform was configured to restrict responses to one entry per unique IP address and required mandatory validation fields. Concurrently, to mitigate digital response bias and include physicians underrepresented online, the research team conducted in-person, face-to-face recruitment within clinical workplaces. The team targeted major public and private healthcare facilities across primary, secondary, and tertiary tiers. While overall sampling remained bound to non-probability convenience constraints, researchers systematically rotated recruitment times across morning, evening, and night shifts across distinct clinical departments (such as Internal Medicine, Surgery, and Pediatrics) to maximize the diversity and breadth of the clinical cohorts sampled. This dual methodology, digital and personal, greatly enhanced participant engagement, with a response rate of face-to-face interviews approximately 97%, while ensuring data integrity across the various medical specialties that comprise two sections. Section one included sociodemographic characteristics. Section two included the Maslach Burnout Inventory-Human Services Survey (MBI-HSS). The MBI-HSS is a self-administered or interviewed questionnaire consisting of 22 statements divided into three main domains evaluated as the following: for EE (low: < 13, moderate: 14–26, high > 27), for DP (low: < 5, moderate 6–9, and high > 9), and for PA (low > 40, moderate 34–39, high: < 33). Each respondent answered according to their perceived frequency of occurrence, ranging from never to every day (on a corresponding scale from 0 to 6) (15).

### Inclusion and exclusion criteria

To ensure a well-defined study population, strict inclusion and exclusion criteria were applied to all potential participants. The inclusion criteria for this study required participants to be licensed physicians who were actively practicing in either a public, private, or dual-sector healthcare facility (including hospitals, secondary, and primary healthcare centers) across Libya during the study period. Additionally, participants had to be willing and able to provide written informed consent before data collection. Conversely, individuals were excluded from the study if they were medical students or interns, as these groups have not yet achieved full medical licensure or independent practice status. Furthermore, physicians who were on prolonged leave exceeding 3 months or those who had fully retired from clinical practice were excluded to ensure the sample accurately reflected the current, active workforce facing day-to-day occupational stressors in the Libyan healthcare system.

The data were analyzed using IBM SPSS software version 25.0. Continuous variables are represented as the mean and standard deviation (±SD), and categorical variables are defined as frequency and percentage. The Cronbach’s Alpha for the MBI-HSS in this study was 0.735, indicating acceptable reliability. The ANOVA test was used to compare more than two groups. An analysis was considered significant if the *p*-value was <0.05.

To identify independent predictors of severe psychological strain and control for potential confounding factors, a multi-stage modeling approach was employed. First, univariable (bivariate) logistic regression analyses were performed for all demographic and work-related characteristics. Variables demonstrating a statistical association at a threshold of *p* < 0.05 in the bivariate screening, or those identified as clinically vital in the existing literature, were subsequently entered into the final multivariate models.

For the multivariate phase, three separate binary logistic regression models were constructed—one for each primary dimension of the MBI-HSS treated as a dichotomized outcome (High vs. Low/Moderate) based on standard diagnostic cut-off thresholds. While continuous linear models capture total score variance, dichotomization was intentionally selected to isolate the specific subset of physicians experiencing severe, clinically relevant burnout levels and to facilitate direct comparison with international epidemiological literature.

All selected predictors were entered simultaneously into their respective models using the standard entry method (SPSS version 25.0). Categorical variables were converted to dummy variables, utilizing designated baseline reference categories to calculate comparative adjusted risks: the 25–35 years cohort for age group, Married for marital status, Private sector for workplace environment, and Pathologist for medical specialty. Final results were expressed as adjusted Odds Ratios (aOR) alongside their corresponding 95% Confidence Intervals (CI), with statistical significance established *a priori* at a two-tailed *p*-value < 0.05.

## Ethical consideration

Written informed consent was obtained from each study participant before data collection. Moreover, the study was approved by the Ethics Committee (Standing Committee for Biosafety and Bioethics, NBC: 002. H-23.17). The protocol was previously published, and the study was carried out according to the Helsinki Declaration (16).

## Results

### Socio-demographic and work-related characteristics

**Figure 1:**
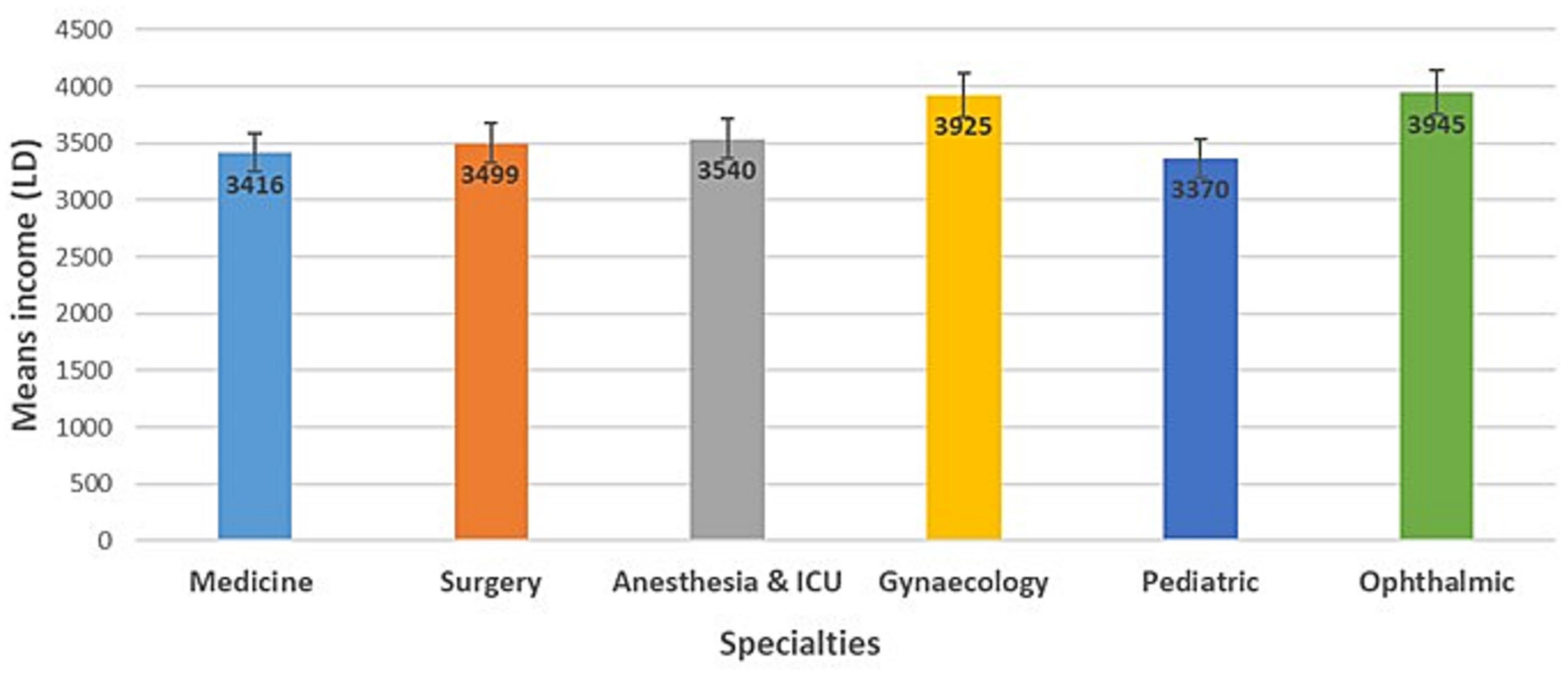
Means with (SD) income in various specialties with Libyan Dinar (LD).

A total of 731 participants were recruited from healthcare facilities (hospitals, secondary healthcare, and primary healthcare) across three regions of Libya: western (37%), eastern (35%), and southern (28%). No statistically significant differences were found in recruitment across the study period (March–September 2023). Demographic information was included; mean age (± SD) 36.76 years old (± 7.23), number of males and females 284 (38.9%) 447 (61.1%) respectively, number of single 270 (36.9%), married 444 (60.7%), divorced 13 (1.8%), and widow 4 (0.5%), mean income 1,672 (±2177.19) Libyan Dinar (LD); $344.74 (± 448.90) Dollar (US), and mean vacation 16.85 ± 26.03 days per year (Figure 1; Tables 1, 2).

**Table 1:** Sociodemographic and work-related characteristics of respondents.

Sociodemographic	Number	%
Age		
25–35	373	51.0
36–45	285	39.0
>46	73	10.0
Mean ± SD	36 ± 7.23	
Sex		
Male	284	38.9
Female	447	61.1
Marital status		
Single	270	36.9
Married	444	60.7
Divorced	13	1.8
Widow	4	0.5
Vacation days in the last 12 months		
0–30 days	651	89.1
31–60 days	48	6.6
61–90 days	17	2.3
>91 days	15	1.5
Mean ± SD	16.85 ± 26.03	
Income Mean ± SD	1,672 (±2177.19)LD 344.74 (± 448.90) US	Currency exchange 4.85 US
Specialist		
Medicine	223	30.5
Surgery	111	15.2
ICU and anesthesia	127	17.4

**Table 2:** The number and percentage of specialists’ response and their work type.

Sociodemographic	Number	%
Obstetrics and gynecology	86	11.8
Pediatric	71	9.7
Radiology	20	2.7
Ophthalmology	40	5.5
Dermatology	31	4.2
ENT	7	1.0
Community medicine	7	1.0
Pathology	8	1.1
Place of work		
Public	562	76.9
Private	53	7.3
Both	116	15.9

Our study involved physicians from different specialties, as shown in (Tables 1, 2), and included Medicine 223, Surgery 111, ICU and Anesthesia 127, Obstetrics and Gynecology 86, Pediatrician 71, Radiology 20, Ophthalmology 40, Dermatology 31, ENT 7, Community Medicine 7, and Pathologist 8. Where 562 (76.9%) physicians worked in public hospitals, 53 (7.3%) physicians worked in private hospitals, and 116 (15.9%) physicians worked in both.

### Prevalence of burnout among physicians

The distribution of the Maslach Burnout Inventory-Human Services Survey (MBI-HSS) subscale scores revealed varying degrees of severity across the three dimensions (Table 3). For the Emotional Exhaustion (EE) subscale, 108 physicians (14.8%) scored low, 279 (38.2%) fell within the moderate range, and 344 (47.1%) reported high levels of exhaustion. Regarding Depersonalization (DP), none of the participants scored in the low category; 100 physicians (13.7%) demonstrated a moderate level, while the vast majority (*n* = 631, 86.3%) exhibited high depersonalization scores. Lastly, evaluation of the Personal Accomplishment (PA) subscale showed that 478 physicians (65.4%) reported low feelings of accomplishment—reflecting severe burnout risk in this domain, whereas 117 (16.0%) and 136 (18.6%) reported moderate and high scores, respectively (Table 3).

**Table 3:** The frequency of physicians by levels of burnout in the three dimensions.

Burnout dimensions	Low
Emotional exhaustion^a^	
Low	108 (14.8%)
Moderate	279 (38.2%)
High	344 (47.1%)
Depersonalization^b^	
Low	0
Moderate	100 (13.7%)
High	631 (86.3%)
Personal accomplishment^c^	
Low	478 (65.4%)
Moderate	117 (16.0%)
High	136 (18.6%)

^a^Score of ≤16 denoted a low level, a score of 17 to 26 denoted a moderate level, and a score ≥27 denoted a high level. ^b^Score of ≤ 6 denoted a low level, 7 to 12 denoted a moderate level, and a score of ≤13 denoted a high level. ^c^Score of ≥ 39 indicated a low level, 32 to 38 denoted a moderate level, and ≤31 denoted a high level.

### Prevalence of burnout among physicians’ specialties

The results revealed the highest burnout rates among the medicine, 16.8%; ICU and Anesthesia 10.0%; surgery 7.6%; obstetrics and gynecology, 5.3%; and pediatrics, 4.2% (Table 4).

**Table 4:** The frequency of physicians according to specialties.

Specialties	Section-A	*p*-value	Section-B	*p*-value	Section-C	*p*-value	Total
Medicine	122 (16.6%)	0.00	205 (28.0%)	0.00	43 (2.1%)	0.289	16.8%
ICU and Anesthesia	75 (10.2%)		114 (15.5%)		32 (4.3%)		10.0%
Surgery	57 (7.7%)		93 (12.7%)		18 (2.4%)		7.6%
Obstetrics and gynecology	24 (3.2%)		76 (10.3%)		17 (2.3%)		5.3%
Pediatrics	27 (3.6%)		57 (7.7%)		9 (1.2%)		4.2%

### Bivariate associations with burnout dimensions

In the main results, unadjusted bivariate analysis was conducted to evaluate the relationship between socio-demographic and work-related characteristics and high levels of the three MBI-HSS subscales (Tables 5, 6).

**Table 5:** Parameters associated with burnout syndrome and its dimensions among physicians.

Sociodemographic Characteristic and work-related burnout	*N*	Emotional Exhaustion	Depersonalization	Personal accomplishment
		Mean ± SD	Mean ± SD	Mean ± SD
Age				
25–35	373	2.35 ± 0.69	2.35 ± 0.69	1.62 ± 0.822
36–45	285	2.36 ± 0.72	2.26 ± 0.772	1.48 ± 0.75
>46	73	1.98 ± 0.75	1.98 ± 0.754	1.24 ± 0.64
*p*-value		0.001	0.001	0.001
Sex				
Male	284	2.39 ± 0.66	2.61 ± 0.14	1.58 ± 0.83
Female	447	2.27 ±. 74	2.86 ± 0.34	1.49 ± 0.75
*p*-value		0.032	0.974	0.153
Marital status				
Single	270	2.43 ± 0.674	2.88 ± 0.31	1.57 ± 0.81
Married	444	2.25 ± 0.737	2.85 ± 0.35	1.49 ± 0.76
Divorced	13	2.38 ± 0.767	2.84 ± 0.37	1.92 ± 0.86
Widow	4	2.00 ± 717	2.75 ± 0.50	2.00 ± 1.15
*p*-value		0.011	0.083	0.086
Vacation days in the last 12 months				
0–30 days	651	2.32 ± 0.709	2.86 ± 0.338	1.53 ± 0.792
31–60 days	48	2.22 ± 0.778	2.79 ± 0.410	1.47 ± 0.743
61–90 days	17	2.58 ± 0.618	2.88 ± 0.332	1.76 ± 0.903
>91 days	15	2.20 ± 0.941	2.86 ± 0.351	1.33 ± 0.617
*p*-value		0.309	0.522	0.449
Specialist				
Medicine	223	2.41 ± 0.710	2.91 ± 0.273	1.52 ± 0.798
Surgery	111	2.40 ± 0.679	2.83 ± 0.370	1.45 ± 0.759
ICU and anesthesia	127	2.51 ± 0.640	2.89 ± 304	1.70 ± 0.846
Obstetric and gynecology	86	2.10 ± 0.669	2.88 ± 0.322	1.60 ± 0.801
Pediatric	71	2.14 ± 0.780	2.80 ± 0.400	1.40 ± 0.708
Radiology	20	2.60 ± 0.598	3.00 ± 0.001	1.80 ± 0.833
Ophthalmology	40	2.00 ± 0.716	2.75 ± 0.438	1.30 ± 0.648
Dermatology	31	2.12 ± 0.763	2.67 ± 0.475	1.38 ± 0.715
ENT	7	1.85 ± 0.899	2.71 ± 0.487	1.71 ± 0.951
Community medicine	7	2.42 ± 0.534	2.71 ± 0.487	1.42 ± 0.786
Pathology	8	1.50 ± 0.534	2.62 ± 0.517	1.37 ± 0.744
*p*-value		0.001	0.001	0.059

**Table 6:** Parameters associated with burnout syndrome and its dimensions among place work.

Sociodemographic characteristic and work-related burnout	*N*	Emotional Exhaustion	Depersonalization	Personal accomplishment
		Mean ± SD	Mean ± SD	Mean ± SD
Place of work				
Public	562	2.33 ± 0.71	2.85 ± 0.35	1.55 ± 0.57
Private	53	1.96 ± 0.73	2.79 ± 0.40	1.22 ± 0.79
Both	116	2.43 ± 0.70	2.93 ± 0.23	1.56 ± 0.83
*p*-value		0.001	0.015	0.013

The Age represented a highly significant factor across all three burnout dimensions, showing strong statistical associations with high Emotional Exhaustion, high Depersonalization, and low Personal Accomplishment (all *p* < 0.001). Also, Practice specialty was significantly associated with both Emotional Exhaustion and Depersonalization (*p* < 0.001). However, its association with low Personal Accomplishment did not reach statistical significance (*p* = 0.059). The healthcare sector where physicians practiced was found to be a significant factor uniquely associated with high Emotional Exhaustion (*p* < 0.001).

The marital status did not reach the threshold for statistical significance for any subscale. It demonstrated only marginal, non-significant trends toward higher depersonalization (*p* = 0.083) and lower personal accomplishment (*p* = 0.086; Tables 5, 6).

### Multivariate logistic regression analysis of predictors for burnout dimensions

To identify independent risk factors for high levels of burnout among Libyan physicians, a multivariate logistic regression analysis was performed to estimate Adjusted Odds Ratios (aORs) with 95% Confidence Intervals (CIs) across all three MBI-HSS dimensions (Table 7).

**Table 7:** Multivariate logistic regression analysis of independent predictors for burnout dimensions.

Predictor variables	Emotional exhaustion (EE)aOR (95% CI); *p*-value	Depersonalization (DP)aOR (95% CI); *p*-value	Low personal accomplishment (PA)aOR (95% CI); *p*-value
Age group			
25–35 years	Ref	Ref	Ref
36–45 years	0.98 (0.72–1.34); *p* = 0.350	0.96 (0.65–1.42); *p* = 0.410	0.85 (0.58–1.24); *p* = 0.290
> 46 years	0.42 (0.21–0.85); ***p* < 0.001**	0.38 (0.15–0.92); ***p* < 0.001**	0.31 (0.12–0.78); ***p* < 0.001**
Marital status			
Married	Ref	Ref	Ref
Single/Unmarried	1.12 (0.84–1.48); *p* = 0.412	1.85 (0.91–3.42); *p* = 0.083	1.62 (0.93–2.95); p = 0.086
Workplace sector			
Private sector	Ref	Ref	Ref
Public healthcare	2.45 (1.62–3.71); ***p* < 0.001**	2.10 (1.25–3.53); ***p* = 0.015**	1.95 (1.18–3.22); ***p* = 0.013**
Dual employment (Both)	2.88 (1.91–4.15); ***p* < 0.001**	2.95 (1.44–5.21); ***p* = 0.015**	1.74 (1.02–2.98); ***p* = 0.041**
Medical specialty			
Medicine (*n* = 223)	Ref	Ref	Ref
ICU and anesthesia (*n* = 127)	1.23 (0.88–1.72); *p* = 0.210	0.96 (0.61–1.51); *p* = 0.870	1.56 (1.01–2.41); *p* = 0.044
Surgery (*n* = 111)	0.45 (0.28–0.73); ***p* = 0.001**	0.48 (0.26–0.88); ***p* = 0.018**	0.82 (0.51–1.32); *p* = 0.410
Obstetrics and gynecology (*n* = 86)	0.31 (0.18–0.54); ***p* < 0.001**	0.35 (0.18–0.68); ***p* = 0.002**	0.76 (0.44–1.31); *p* = 0.320
Pediatrics (*n* = 71)	0.25 (0.13–0.48); ***p* < 0.001**	0.22 (0.10–0.48); ***p* < 0.001**	0.64 (0.35–1.17); *p* = 0.145
Pathologist (*n* = 8)	0.32 (0.11–0.94); ***p* = 0.038**	0.24 (0.06–0.89); ***p* = 0.033**	0.65 (0.21–2.04); *p* = 0.462
Other Subspecialties (*n* = 105)	0.34 (0.21–0.55); ***p* < 0.001**	0.31 (0.17–0.56); ***p* < 0.001**	0.71 (0.45–1.12); *p* = 0.138

Bold values indicates (EE): Emotional exhaustion; (DP): Depersonalization; (PA): Low personal accomplishment; (aOR): Odds Ratio; (*n*)= Number.

#### Independent predictors of emotional exhaustion

The multivariate model revealed that the working environment significantly impacts this dimension. Compared to their private-sector counterparts, physicians operating in the public sector exhibited a 2.45-fold higher risk for high Emotional Exhaustion (aOR = 2.45, 95% CI: 1.62–3.71, *p* = 0.001), while dual-sector employment in both public and private settings yielded an even higher independent risk (aOR = 2.88, *p* = 0.001).

In terms of clinical specialties, ICU & Anesthesia did not show a statistically significant difference from the Medicine reference group. However, Surgery emerged as a significant protective factor, demonstrating much lower odds of experiencing high Emotional Exhaustion (aOR = 0.45, 95% CI: 0.28–0.73, *p* = 0.001) relative to Medicine. Additionally, older age emerged as a highly significant protective factor; physicians aged over 46 years were 58% less likely to suffer from high emotional exhaustion (aOR = 0.42, 95% CI: 0.21–0.85, *p* = 0.001) compared to the younger 25–35 years cohort.

#### Independent predictors of depersonalization

Systemic sector pressures and demographics heavily predicted detached physician attitudes. Public healthcare facility placement significantly increased the independent risk, showing a 2.10-fold higher risk for high Depersonalization (aOR = 2.10, 95% CI: 1.25–3.53, *p* = 0.015). Dual-sector practitioners again faced an elevated risk profile (aOR = 2.95, *p* = 0.015). Surgical subspecialties maintained a lower risk profile compared to internal medicine, presenting significantly lower odds of high Depersonalization (aOR = 0.48, 95% CI: 0.26–0.88, *p* = 0.018). Conversely, older physicians (≥ 46 years) were 62% less likely to experience high depersonalization (aOR = 0.38, 95% CI: 0.15–0.92, *p* = 0.001) than their younger peers. Single marital status did not achieve strict statistical significance but showed a marginal trend effect associated with an elevated risk for high Depersonalization (aOR = 1.85, *p* = 0.083).

#### Independent predictors of diminished personal accomplishment

Workplace conditions and early career status were the prominent features impacting low professional efficacy. Public sector employment significantly increased the risk for low Personal Accomplishment by 1.95-fold (aOR = 1.95, 95% CI: 1.18–3.22, *p* = 0.013) compared to private practice. Within the clinical cohorts, a statistically significant elevation in the risk of low Personal Accomplishment was observed specifically within the ICU & Anesthesia cohort (aOR = 1.56, 95% CI: 1.01–2.41, *p* = 0.044) relative to the Medicine reference group. Protective features were again dominated by age parameters, with physicians over 46 years being 69% less likely to experience a diminished sense of personal accomplishment (aOR = 0.31, 95% CI: 0.12–0.78, *p* = 0.001). Finally, unmarried/single status demonstrated a marginal, non-significant trend effect on low Personal Accomplishment (aOR = 1.62, *p* = 0.086).

## Discussion

To the best of our knowledge, this is the first study to investigate the prevalence of burnout across all medical specialties among Libyan physicians. Previous literature from the post-COVID-19 era (2020–2022) was restricted to localized data, such as a single internal medicine department in Benghazi. By contrast, our study provides a broader, multi-specialty evaluation of the systemic psychological strain affecting the country’s medical workforce.

The subscale analysis revealed high occupational strain among Libyan physicians. Severe scores were prominent in the emotional and behavioral domains, with 47.1% exhibiting high Emotional Exhaustion (EE) and 86.3% showing high Depersonalization (DP). Crucially, professional fulfillment was heavily compromised, as most of the cohort (65.4%) reported low scores for Personal Accomplishment (PA), reflecting severe burnout risk in this domain—while only 18.6% maintained a high, protective level of personal achievement. Multivariate analysis identified age, medical specialty, and workplace sector as significant independent risk factors for severe physician burnout. Conversely, marital status did not demonstrate a statistically significant relationship with increased burnout levels.

The severe occupational strain observed among Libyan physicians in this study aligns with, and in some domains exceeds, global and regional trends reported in recent literature. For instance, our finding that high Emotional Exhaustion (EE) and Depersonalization (DP) are prominent among early-career physicians is consistent with international data. Ferguson et al. similarly identified high rates of EE (47.8%) and DP (29.0%) among resident physicians across all specialties in Canada, noting that younger age and lower financial resources independently magnified this vulnerability (17). Regionally, our data mirror the severe levels of behavioral and psychological distress reported in neighboring Arab countries, though with distinct institutional variations. A study among resident physicians in Tunisia revealed a similarly profound psychological burden, where 70.7% experienced high emotional exhaustion and 44.2% exhibited high depersonalization (18). Similarly, research among middle-grade physicians in Saudi Arabia documented high levels of exhaustion (68.8%) and depersonalization (63.6%), driven primarily by unmarried status and fewer years in active clinical service (19). However, a critical divergence emerges regarding professional efficacy. While the Sudanese cohort reported a high level of decreased professional achievement (73.1%) (10), only 38.5% of Saudi physicians reported low personal accomplishment. In comparison, our cohort demonstrated a significantly compromised professional efficacy domain, with 65.4% of Libyan physicians reporting low (PA). This suggests that the structural and post-conflict infrastructural challenges within the Libyan healthcare sector may erode a clinician’s sense of professional fulfillment more severely than the institutional environments in wealthier regional counterparts like Saudi Arabia. Furthermore, while Western and Latin American literature—such as recent data from Brazil—frequently attributes severe burnout (59.4%) to individualized stress factors like a lack of personal hobbies and work–family conflicts (20), our multivariate models show that systemic institutional placement, specifically operating within public healthcare facilities, remains the overriding independent driver of multi-dimensional burnout among Libyan practitioners.

In addition, the AMA determined the prevalence of burnout among American physicians’ specialties. The results reported that the highest burnout rates were among those in Urology 54%, Neurology 50%, Nephrology 49%, Diabetes and Endocrinology 46%, Family medicine 46%, and Radiology 46%. The lowest rates of burnout were among public health and preventive medicine (29%), Ophthalmology (30%), orthopedics (34%), Psychiatry (35%), Otolaryngology (35%), and General surgery (35%) (21). These variations could be due to differences in workplace culture, the nature of the health system, the responsibilities and roles expected of physicians, the volume of patients treated by a single physician, as well as poor employment conditions in Libya.

According to the World Bank (22), Libya had 2.09 physicians per 1,000 people in 2022, while the Organization for Economic Co-operation and Development (OECD) average was 3.6 per 1,000 people in 2019 (23). This suggests that Libyan physicians experience higher pressure due to the drop in the number of physicians per 1,000 people. The main reason for this was related to two factors, including education and economic factors (24).

In the same context, purchasing power parity (PPP) is a crucial economic indicator that refers to the quantity of goods and services that can be purchased with a specific amount of currency (25). In 2023, the PPP conversion factor was 1.82, which means that 1.82 Libyan dinars is equivalent to 1 US dollar in terms of purchasing power. This indicates that the cost of goods and services in Libya is 82% higher compared to the United States (26). Taking into consideration the mean income of 1,672 (±2177.19) Libyan Dinar (LD), $344.74 (± 448.90) Dollar (US). Many physicians may find their salaries insufficient to cover the rising costs of goods and services.

Therefore, economic instability and inadequate salaries can lead to brain drain, where skilled healthcare professionals leave Libya for better opportunities abroad. This exodus further strains the healthcare system, leading to a shortage of qualified medical staff (24).

Indeed, gender can play a significant role in the experience of burnout. In our study, women constituted 61.1% of the participants. A female physician often faces unique challenges, including maintaining a balance between work and family responsibilities, particularly with regard to childcare, social pressures, and workplace discrimination. These factors contribute to a greater sense of burnout (27).

Although our survey did not directly measure the domestic or clinical consequences of burnout, established literature suggests that severe occupational strain can yield significant knock-on effects. Professionally, high emotional exhaustion and depersonalization are documented to erode empathy, potentially compromising the physician-patient dynamic, lowering patient satisfaction, and increasing the risk of medical errors (28, 29). Conversely, implementing individual-level stress reduction initiatives—such as physical conditioning and interactive coping programs—has been shown to significantly decrease clinical errors within 6 to 12 months (28–31). Beyond the clinic, chronic exhaustion may spill over into personal lives, contributing to social isolation and heightened familial conflict (28).

Moreover, the multivariate logistic regression analysis provided significant insights, identifying workplace setting and subspecialty as the primary independent predictors of physician burnout in Libya. Notably, even after adjusting for all demographic variables, working in the underfunded public health sector or in high-acuity fields independently increased the likelihood of severe emotional exhaustion and depersonalization, with the ICU & Anesthesia specialty further demonstrating a significantly higher independent risk for low Personal Accomplishment. Conversely, the model identified older age as a strong protective factor, with senior clinicians showing much lower risks across all burnout dimensions than early-career doctors. These findings demonstrate that the physician burnout crisis in Libya is heavily influenced by institutional and structural characteristics, which emerged as powerful independent predictors in our models. While our data do not account for unmeasured individual psychological traits or personal resilience factors, the strong statistical associations found here highlight an urgent need to prioritize targeted systemic reforms within the healthcare infrastructure.

This deep reliance on structural drivers in Libya stands in stark contrast to the patterns observed in high-resource Western settings, where burnout fluctuations are often tied to shifts in individual personal time and administrative burdens. For context, longitudinal data from the American Medical Association (AMA) demonstrated that the prevalence of burnout among U. S. physicians fluctuated from 45.5% in 2011 to 43.9% in 2017—a decline largely attributed to improvements in physicians’ personal time and modified institutional workflows (32). While these Western trends suggest that burnout can be mitigated by adjusting personal time metrics, our findings from Libya indicate a more deeply rooted crisis. In a post-conflict healthcare system, minor personal or individual-level adjustments are insufficient; rather, the overwhelming occupational strain is driven by fundamental systemic and infrastructural deficiencies within the public sector that require macro-level institutional reforms.

Moreover, based on our findings, targeted reforms are urgently required within the Libyan healthcare system. This includes implementing mandatory workload limits and structured scheduling to reduce public-sector and dual-employment strain, establishing formal peer-led mentorship and confidential counseling networks to protect vulnerable early-career physicians (aged 25–35), and creating clear promotional pathways alongside ensuring stable access to essential medical supplies to restore professional efficacy within high-acuity units like the ICU and anesthesia.

## Limitations

While this study offers valuable and unique insights into the prevalence and independent predictors of burnout syndrome among Libyan physicians, several limitations should be noted. First, the cross-sectional design captures data at only one point in time, preventing us from establishing direct causal links between risk factors. Second, lacking an updated, centralized registry of active physicians in post-conflict Libya meant we had to rely on a non-probability convenience sampling method instead of a randomized approach; Consequently, while our data highly reflects the multi-specialty workforce within major urban and regional medical centers, caution should be exercised when generalizing these findings to highly remote or understaffed rural clinical settings across Libya. Third, despite supplementing the survey with face-to-face workplace interviews to boost engagement, the online questionnaire distributed via Kobo Toolbox might introduce selection bias, possibly favoring more tech-savvy clinicians or those with stable internet access. Lastly, burnout was assessed using self-report questionnaires, which may be influenced by social desirability and recall biases. However, this was mitigated by using a culturally and linguistically validated Arabic version of the MBI-HSS, which showed acceptable internal reliability (Cronbach’s Alpha = 0.735) in our sample.

## Conclusion

In conclusion, this research highlights that physician burnout is a significant public health issue in Libya’s healthcare system, characterized by high emotional exhaustion, profound depersonalization, and severely compromised professional fulfillment. Our findings demonstrate that this occupational strain is strongly associated with structural and institutional factors—specifically affecting public-sector facilities and high-acuity specialties—rather than individual factors. Accordingly, mitigating this systemic strain requires prioritizing institutional interventions. Implementing macro-level structural reforms, such as improving workplace safety, optimizing departmental workflows, and addressing deficiencies in foundational infrastructure, is essential to preserving the stability of the medical workforce and safeguarding the quality of patient care nationwide.

## Data Availability

The original contributions presented in the study are included in the article/supplementary material, further inquiries can be directed to the corresponding author/s.

## Footnotes

1
https://ee.kobotoolbox.org/x/mt7htocK
